# COVID-19 and the Growth Potential

**DOI:** 10.1007/s10272-021-0950-4

**Published:** 2021-01-26

**Authors:** Michael Grömling

**Affiliations:** Gesamtwirtschaftliche Analysen und Konjunktur, German Economic Institute, Konrad-Adenauer-Ufer 21, 50668 Köln, Germany

## Abstract

The lasting economic impact of the coronavirus pandemic will become apparent in the development of the macroeconomic factors of production — labour, capital, human capital as well as the stock of technical knowledge. Changes in behaviour such as a greater acceptance of technology can strengthen potential output permanently. By contrast, negative effects may arise from growing protectionist attitudes or long-lasting uncertainties and ‘scarring effects’. In any case, the coronavirus crisis has induced a technology push. This may be intensified if digitisation gains additional support from investments in infrastructure or if the pandemic heralds a renaissance in the natural sciences — with a corresponding impact on human and physical capital as well as on technical knowledge. For the time being, it is unclear what effects the restructuring and secular structural change will have on potential output. However, dangers are lurking in the acceleration of geopolitical tensions, a misunderstanding of technological sovereignty and increasing government interventions, which, as a whole, could hamper innovation and investment.

The coronavirus pandemic turned into a historical social and economic challenge over the course of 2020. The public health policy measures and the diverse economic impact around the world impeded economic life more than any event in the last seven decades. In 2020, the global economy experienced its sharpest decline during this timeframe. Due to the broad impact of this economic crisis, the macroeconomic effects in Europe will be at least on the same scale as the financial market crisis in 2009.

In addition to this historic economic slump caused by the pandemic, the question also arises as to what lasting effects seem possible. In the following article, some potential implications for advanced economies are discussed (see also Grömling, 2020a), focusing primarily on the effects that the COVID-19 pandemic could have on macroeconomic potential output. Beyond that, the coronavirus crisis will affect public finances (Gros, 2020) and the distribution of income and wealth. In this context, the question arises of how inclusive the structural change that could be triggered by the pandemic may be for the various socio-economic groups in society (Grömling and Klös, 2019; Eichhorst et al., 2020). These issues are not addressed here.

**Michael Grömling**, German Economic Institute (IW), Cologne, Germany.

The production potential can be described in terms of the endowment of an economy with labour, physical capital (including infrastructure and intangible assets), human capital, natural capital and the diverse stock of technical knowledge (see Figure 1). This factor endowment determines the overall economic productivity level, which in turn determines to a large extent the (material) standard of living in a country. The propensity to invest in all these factors is ultimately governed by the institutional and geopolitical framework.

### Positive and negative behavioural effects

The coronavirus pandemic hit economies more quickly, more intensively and above all more broadly than previous crises. With the abrupt, in part even complete discontinuation of normal business operations in the second quarter of 2020, new technical and organisational alternatives were quickly adopted – such as working or studying from home. Previous resistance to technological solutions has obviously been overcome and potentially created a greater openness to innovation in business and social life.

Macroeconomically, these changes in behaviour can permanently increase both human capital and the stock of technological knowledge and possibly also stimulate higher economic growth in the future. Last but not least, this can be reinforced if young people are influenced positively in their educational decisions and risk awareness. It is conceivable, for example, that they might have a greater affinity for careers in technical fields and the natural sciences.

By contrast, however, there could also be changes in behaviour that hamper a country's progress in the long term

Figure 1
**Production factors as a reference framework for the coronavirus shock**

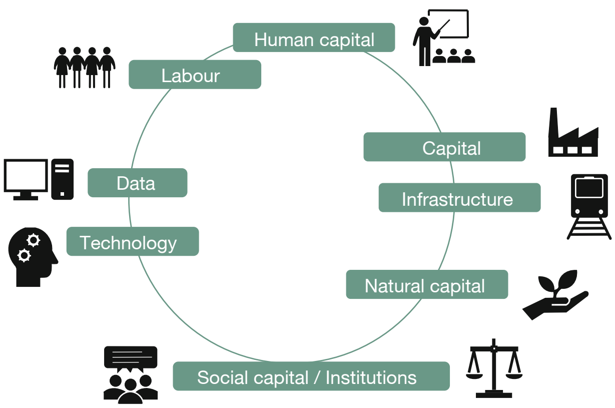

Sources: Author's illustration.

with regard to production factors. If young people are frustrated by limited options for learning, studying and working due to the pandemic, there may be protracted damages to potential output in terms of reduced labour market integration or lower incentives for building up human capital (Maguire, 2020; Tamesberger and Bacher, 2020). According to the so-called scarring effect, young people whose employment has been delayed or who are unemployed due to the recession will experience long-term consequences such as CV gaps and lower total lifetime earnings (Möller and Umkehrer, 2015; Hutter and Weber, 2020).

Kozlowski et al. (2020) show how a tail risk – here with reference to the coronavirus pandemic – can change long-term behaviour, which will then have a negative impact on economic development. Such tail events lead to the aforementioned scarring effects for investors. Companies will take the pandemic into account when making future investment decisions – even if it can be combatted with a vaccine. If the pandemic leads to lower returns on capital in the short term, future returns on capital will also be estimated against this backdrop. This 'scarring of beliefs' would then dampen the propensity to invest over the long term and thus the development of potential output.

Another conceivable possibility for long-term potential damage due to the COVID-19 pandemic is that it will intensify the geopolitical risks and protectionist attitudes that predate the crisis. This can inhibit the cross-border allocation of labour and the international transfer of knowledge – for example by limiting options and reducing incentives for training and work experience abroad. If the pandemic and a less open global economy lead to a general reduction in the migration of skilled labour over the long term, there will be a lower production potential due to the expected demographic development – i.e. the shrinking and ageing of populations in advanced economies. Finally, a growing anti-market attitude as a result of the pandemic would also adversely affect economic life and structural change.

## Boost for technological progress

The potential positive effects on human capital and the stock of technology have already been discussed in the context of the outlined behavioural changes. The digitisation of the economy should enjoy another long-term boost as a result of the pandemic (Klös, 2020). To compensate for the restrictions on labour input due to the lockdown, companies and private households have invested in technical equipment. This capital stock and the intangible components in particular, such as organisational capital, will continue to be available in the future (see Grömling, 2020b). To some extent, specific measures of the comprehensive economic stimulus packages passed by many governments also promote technological progress. Experiences from the financial market crisis in 2008 and 2009 indicate that a portion of the crisis-related underemployed research staff at that time used their working hours to improve the existing production and organisation processes (Rammer, 2011). This has increased intangible assets and the level of technical knowledge.

Beyond the short-term necessities, this pandemic-related technology push may be intensified if further and continuous technological improvements are now made in public infrastructure. Last but not least, the crisis has revealed the great potential – for example in schools, the health care system or public administration – for the digitisation of services and production processes. The current crisis can accentuate pre-existing needs for public investment (Hüther and Bardt, 2020) and stimulate a corresponding accumulation of capital.

The dependencies that arose in some areas of the economy – for example, through a lack of foreign or domestic intermediates in both the manufacturing and the service sector (Grömling, 2020c) – have increased the pressure to search for alternatives. Over the long term, this should strengthen companies' resilience. A restructuring of production processes, such as the relocation of value-added components, can raise companies' production costs on the one hand, while creating corresponding incentives for increasing capital formation, e.g. through additional automation, on the other.

Finally, the significance of the natural sciences has been revived, but not only because of the current crisis. The potential for the corresponding development of human, physical and technological capital have already been mentioned before with regard to the major global megatrends – such as the global population growth, the scarcity of resources and climate change (Grömling, 2019). In all the natural sciences – a specific example being research on antibiotics – the current pandemic can boost an advance in technology and increase investment.

Over the long term, it is conceivable that the coronavirus pandemic will have positive effects on macroeconomic output due to an increase in technical knowledge. However, companies' financing situations for such investments must also be taken into account. For epidemic reasons, the crisis is currently connected with uncertainties that are difficult to assess, e.g. regarding the recovery prospects of the world economy. The global economy and growth prospects are of great significance for economies that are highly integrated into the international division of labour and global trade – for example, German manufacturing firms generate roughly half of their turnover from foreign customers. High levels of uncertainty will dampen the propensity to invest. The financing options for companies are also impaired by the tense economic situation. This applies to both raising equity capital and financing with debt (Demary and Hüther, 2020). Declining turnover and profit are putting a strain on the companies' equity base. Increasing risks of insolvency curb the granting of loans by banks and raise investment costs through higher risk premia. However, most governments' stimulus packages include measures to stabilise corporate financing. After all, technical progress also depends on the establishment of new companies – the coronavirus pandemic has made this very clear. In the area of biotechnology, for example, venture capital becomes more important in order to develop production potential.

### Restructuring and structural change

When the coronavirus epidemic began in China, worries about production restrictions quickly arose due to the loss of intermediates from other countries. This concern did abate in Germany over time (Grömling, 2020c). But supplier dependencies became noticeable and may give rise to the restructuring of supply and production processes along very stretched-out international value-added chains. On the one hand, this can lead to a multi-supplier strategy that is used to eliminate dependencies on individual suppliers and to rediversify risks. On the other hand, the existing geopolitical risks may also lead to a regional reorientation. Higher costs resulting from relocations, a higher level of in-house production or a broader-based inventory management can drive the automation and technological progress.

Ultimately, the advantages of the previous specialisations within the framework of the existing value-added chains will be compared with the benefits of restructuring and new risk diversification. It should always be borne in mind that the previous internationalisation in the form of transnational value-added chains has increased the productivity and competitiveness of companies as a whole and also reduced previously existing risks and dependencies. The impact that a restructuring of international production networks will have on the stocks of human capital, capital and technology is not yet clear. It is also unclear what impact a reorganisation of suppliers will have on the international and mutual transfer of knowledge and technology and the respective factors of production in the previously involved and potentially new partner countries. It is conceivable that technological sovereignty could be defined in the context of the European Union and that this could be seen as a reference framework for the national competencies and for access to the resources, competencies and inputs of partners (Edler et al., 2020).

Furthermore, it is also possible that the pandemic will accelerate the secular structural change (Hüther and Bardt, 2020; Hutter and Weber, 2020). Consideration should be given to the effects of digitisation, decarbonisation and demographic change. There are very diverse explanations for secular structural changes, and all social, political and economic influences are reflected in the economic structure (Grömling, 2011; 2020d). Changes in consumption and the underlying shifts in preferences in a society can be mentioned as a central driver for this secular structural change in production. The increasing complexity of modern societies stimulates the demand for knowledge-intensive services. Changes in production processes and the inter-sectoral and intra-sectoral division of labour can cause companies to concentrate on their core business and offer hybrid products at the same time.

The pandemic has raised the question of which trends of the secular structural change will be reinforced and what impact this may have on the factors of production and the macroeconomic potential output. In regard to social costs, it is also crucial to consider whether the current structural changes will lead to adjustment burdens on the labour market, i.e. structural unemployment (Grömling, 2020d). High unemployment ultimately means that two central factors of production – labour and human capital – are not used adequately and thus opportunities for growth are wasted. Employees with specific qualifications are no longer used in certain economic areas due to changes in preferences or technology, raising the question of whether these qualifications are applicable in other economic activities. If this is not the case, then the employee's flexibility in terms of qualification and training opportunities is crucial. If the gap between the qualifications of the laid-off labour force and the requirements of companies widens over the long term and if this "mismatch" cannot be remedied through training, the risk of structural unemployment and the wasting of human resources will increase. The social system can in turn influence the incentives for education and the search for jobs. Furthermore, the flexibility of the wage system determines the extent and persistence of structural unemployment (Walwei, 2020; Eichhorst et al., 2020).

### Reorientation of market and state

The coronavirus crisis has not only raised the need for rapidly effective stabilisation policy, but has also strengthened the call for industrial policy. While stabilisation policy is primarily aimed at returning the utilisation of the existing production potential to its normal level in the best case, industrial or structural policy measures aim at a change in the level and structure of the production potential (Grömling, 2020d). Structural and industrial policy interventions are also called for in order to cope with the structural changes addressed in the previous section and possibly necessitated by the COVID-19 pandemic. The aim is to actively shape the sectoral structure of the economy and to ensure the survival of companies in certain industries. Given the speed and severity of the current crisis, it is necessary to consider the consequences that a pronounced wave of insolvencies could have for labour and human capital. Unemployment – and particularly structural unemployment – could be accompanied by a permanent devaluation of human capital or at least by pronounced mismatch problems in the labour market.

If certain sectors (e.g. coal, steel, shipyards, automobiles) are dominant for employment and prosperity in certain regions, then structural policy should prevent these regions from lagging behind or becoming impoverished as a result of a structural shock in a specific sector (Hüther et al., 2019). Sectoral structural policy is also justified by allocative market failure: Basic research – for example, on antibiotics or a coronavirus vaccine – can be regarded as a public good. Accordingly, private research has positive external effects for other companies, and thus public research funding prevents a shortage of supply by the market. Furthermore, the design of the economic structure in terms of supporting 'future industries' through research support is brought forward as a justification for industrial policy.

Security policy arguments are also put forward to stabilise an existing economic structure. In strategic areas (e.g. military, health) and critical infrastructures (e.g. communication networks, energy supply), economic dependencies on other countries – and possibly on their state monopolies – should be prevented. In this context, the pandemic has also intensified the demands for national technological sovereignty and thus raised the question of how independent countries should be with regard to so-called critical technologies. This should prevent political dependencies and ensure state sovereignty. Edler et al. (2020) argue that comprehensive technological autocracy is not purposive. Rather, technological sovereignty is about preserving options by building up and preserving one's own competencies and avoiding one-sided dependencies. This is what opens the door to sovereignty in innovation and independent economic development. At any rate, this not-new topic of the appropriate degree of technological sovereignty – particularly in an environment characterised by geopolitical uncertainties – will probably be intensively discussed as a result of the COVID-19 pandemic.

The list of arguments against governmental structural and industrial policy is long (Bardt and Lichtblau, 2020; Grömling, 2020d; SVR, 2009). Subsidising certain industries in order to improve their production conditions always discriminates against the domestic sectors not benefiting from the policy. Moreover, it distorts competition internationally, which may provoke reactions (tariffs or import quotas) from other countries and can hurt prosperity overall as a result of rising transaction costs. An efficient sectoral structural policy requires asymmetries in information: The state must have better knowledge of the supply of goods desired by society than private companies. The current discussion on automotive technologies of the future serves as a good example. The long-term impact of industrial or structural policy on the production factors of a country ultimately depends on whether the economic structures promoted by these policies are competitive and sustainable over the long term. In any case, industrial policy intervention always gives rise to the risk of structural conservation, which can in turn slow innovation and structural change.

As a result of this serious economic crisis, the basic understanding of market and state is being tested. The protectionism that has emerged since the global financial market crisis and the trade conflicts of recent years have already restricted the mechanisms of a market-based coordination. The long-term effects on innovation and investment are likely to be negative. In any case, the former domestic market programmes and world trade rounds are credited with the development of production factors and productivity. Accordingly, increased state intervention and protectionism are likely to have the opposite effect in the long term.

Increased government intervention as a result of the pandemic could lead to constraints on competition and a concentration of power. This is the case when state-owned enterprises and state funds gain increasing influence on economic activity in their own countries and also in other economies. This can have long-term effects on factor accumulation and the secular development of economies (Matthes, 2020). The coronavirus crisis intensifies the political and economic rift between the United States and China. Both are pushing ahead with industrial and structural policy projects that ultimately bring about a reorientation of market and state and force other countries to take action – also institutionally, e.g. with regards to market access or corporate investments. At the end of the day, this is likely to have a negative impact on production potentials, productivity growth and the prosperity that goes with it.

Table 1
**Positive and negative long-term effects of the coronavirus pandemic**

| Positive | Negative |
|---|---|
| Openness to innovation | Scarring effects: education, labour market, investments |
| Push for digitisation | Protectionism / geopolitical tensions |
| Human capital promotion | Structural unemployment |
| Impulses for public infrastructure | Deglobalisation: knowledge transfer / migration |
| Stability of value-added chains | Growing state influence / market criticism |
| Technological sovereignty | Market concentration |
| Risk diversification | |
| Start-up of new companies | |

Sources: Author's illustration.

An increasing concentration of power can also occur in the wake of platform companies. The constraints resulting from the coronavirus pandemic, for example in the area of stationary retail trade, have already strengthened existing alternatives and their business models. These platform companies also enjoyed a higher valuation on capital markets during the crisis. This can create significant financial advantages, especially if the adjustment burdens are drawn out over a longer period of time (Pagano et al., 2020). It could trigger a lasting reallocation of labour, capital, human capital and technological knowledge.

## Conclusions

The coronavirus pandemic has caused an unprecedented global economic bust. At the same time, it will likely accelerate structural changes, which in turn are driven by digitisation, the energy revolution, decarbonisation and demographic changes. Table 1 summarises some of the possible positive and negative effects of the COVID-19 pandemic. This article has identified the possible changes in production potential – with a focus on the production factors and production processes. Ultimately, the institutional framework conditions determine whether and how much is invested in the specific production factors. The international design of those institutions is central to the long-term effects of the coronavirus pandemic.
